# Editorial: Fresh Ideas, Foundational Experiments: Immunology and Diabetes

**DOI:** 10.3389/fendo.2019.00315

**Published:** 2019-05-16

**Authors:** Justin A. Spanier, Hubert M. Tse, Marc S. Horwitz, Brian T. Fife

**Affiliations:** ^1^Department of Medicine, Center for Immunology, University of Minnesota Medical School, Minneapolis, MN, United States; ^2^Department of Microbiology, Comprehensive Diabetes Center, University of Alabama at Birmingham, Birmingham, AL, United States; ^3^Department of Microbiology and Immunology, University of British Columbia, Vancouver, BC, Canada

**Keywords:** type 1 diabetes, autoimmunity, NOD mouse model, type 2 diabetes, islet transplantation

The Fresh Ideas, Foundational Experiments (FIFE): immunology and diabetes research topic is a collection of 13 articles ranging from perspectives, reviews, to hypothesis, and theories all focused on diabetes. The global rise in incidence of Type 1 Diabetes (T1D) does not correlate with genetic drift and indicates that environmental exposures are playing an increasingly significant role. The FIFE:Immunology and diabetes group would like to use this research topic to share their data and ideas to promote collaborations and accelerate the development of novel therapies with the goal of a cure for T1D.

The multidisciplinary FIFE mini-symposium brought together young researchers from across North America investigating various interconnected contributors to T1D onset, progression, interventions, and put forth multiple concepts for further examination. Its members have convened annually for the past 3 years to share their perspective and research updates and establish new collaborations unified by the universally held goal of finding a sustainable, life-long cure. The first perspective in this series by Mouat et al. “Fresh Ideas, Foundational Experiments (FIFE): Immunology and Diabetes 2016 FIFE Symposium” describes the group, its goals, and summarizes the inaugural FIFE mini-symposium held at the University of British Columbia in Canada under the vision and leadership of Dr. Horwitz.

The inaugural FIFE symposium led to 12 additional publications from members of the FIFE collaborative research team. This includes a perspective from Chen et al. entitled “The Role of NOD Mice in Type 1 Diabetes Research: Lessons from the Past and Recommendations for the Future.” The authors describe the usefulness of the non-obese diabetic (NOD) mouse for the past 35 years as a primary model for studying autoimmune diabetes. They focus on the similarities to the human disease, polymorphisms, gene perturbations of a disease that targets similar biological pathways, tissues, and islet antigens. They also address the reasons why immune therapies have failed to translate from mice to humans. Finally, they propose new strategies to edit the NOD genome to improve a better understanding of human diabetes.

With a better understanding of the NOD mouse, we next focus on the complex mechanisms and pathways involved in disease pathogenesis. Our journey begins with a review by Newby and Mathews entitled “Type I Interferon Is a Catastrophic Feature of the Diabetic Islet Microenvironment.” In this review they provide a detailed understanding of the molecular and cellular pathways resulting in islet beta cell destruction. T1D develops from a complex interaction between genetics, the immune system, and environmental factors. The authors focus on type 1 interferons as the link between these critical pieces and review the evidence supporting the diabetogenic potential of IFNα/β within the islet microenvironment for the development of T1D.

At the heart of autoimmune-mediated T cell diseases lies the recognition of self-proteins being presented by the human leukocyte antigen (HLA) complex to the T cell receptor (TCR). The next contribution by Bettini and Bettini address this critical interaction in their review: “Understanding Autoimmune Diabetes through the Prism of the Tri-Molecular Complex.” The strongest susceptibility alleles for T1D reside within the HLA loci, which supports the role for T cells as the critical drivers of T1D. This review provides a summary of autoimmune T cell development, the significance of the antigens targeted in T1D, and the relationship between TCR affinity and immune regulation.

The era of genome-wide association studies (GWAS) has yielded the discovery of ~57 independent loci contributing to the overall genetic risk for T1D development. This next review by Wallet et al. is entitled “Isogenic Cellular Systems Model the Impact of Genetic Risk Variants in the Pathogenesis of Type 1 Diabetes.” In this review, they provide a comprehensive list of single nucleotide polymorphisms associated with T1D risk and summarize the functional impact of several candidate risk variants on host immunity in the context of T1D. They also discuss the potential for an “isogenic disease-in-a-dish model system” to interrogate the biological role of risk variants, with the goal of expediting precision therapeutics in T1D.

The next article in our series is a review by Wagner entitled: “Overlooked Mechanisms in Type 1 Diabetes Etiology: How Unique Costimulatory Molecules Contribute to Diabetogenesis.” CD28 is the classical co-stimulatory molecule while CTLA-4 is the classical inhibitory counterpart. This review is focused on additional co-stimulatory molecules such as TNF-receptors I and II, CD40, mucin, ICOS, and immunoglobulins. Wagner proposes that inflammation driven by interactions between CD40 with CD154 results in the loss of Foxp3 expression and the generation of pathogenic TH40 (CD4+CD40+) effector cells. Thus, targeting the CD40/CD40L pathway creates a potentially new therapeutic avenue for T1D.

The topic for the next review is focused on environmental stressors, namely virus infections, as triggers of T1D in genetically susceptible individuals. The review by Morse and Horwitz, “Innate Viral Receptor Signaling Determines Type 1 Diabetes Onset” focuses on the observation that heritable susceptibility alone cannot explain the rising incidence of T1D. The authors discuss that the recognition of viral antigens via innate pathogen-recognition receptors could trigger inflammatory events which ultimately result in the destruction of insulin-secreting beta cells. They further discuss that activation of innate pathways and inflammatory molecules, including type I and III interferon, can differentially prime the immune system to produce a protective response or a diabetogenic response. The authors conclude by hypothesizing that the increase in incidence of T1D may be due to changes in how the immune system senses and responds to viral antigens.

We close the review sections with discussions of both the immune systems' response to transplanted islets and the health of the islet transplant itself. Barra and Tse discuss “Redox-Dependent Inflammation in Islet Transplantation Rejection.” In this review the authors discuss the main challenges associated with transplant rejection and islet viability, thus preventing long-term β-cell function. Redox signaling and the production of reactive oxygen species (ROS) by recipient immune cells and transplanted islets themselves are key players in the demise of the beta cell and contribute to graft rejection. The authors focus on redox signaling, the process in which ROS are generated during graft rejection as well as new strategies to limit or modulate ROS synthesis during islet cell transplantation.

Transplants containing insulin-producing cells are vulnerable to both recurrent autoimmunity and conventional allograft rejection. Burrack et al.'s review “T Cell-Mediated Beta Cell Destruction: Autoimmunity and Alloimmunity in the Context of Type 1 Diabetes” discuss this complex topic. Current immune suppression acts globally, but ideally, a successful approach would limit T cells targeting the transplanted islets. First, they describe the current understanding of autoimmune destruction of beta cells including the roles of CD4 and CD8 T cells and several possibilities for antigen-specific tolerance induction. Second, they outline diabetic complications necessitating beta cell replacement. Third, they discuss transplant recognition, potential sources for beta cell replacement, and tolerance-promoting therapies under development.

The next review steps outside of the autoimmune field to focus on patients with type 2 diabetes (T2D) as the largest population of patients who experience post-sepsis complications and rising mortality in the review by Frydrych et al. “Diabetes and Sepsis: Risk, Recurrence, and Ruination.” Patients with T2D have an increased risk of developing infections and sepsis. T2D also worsens infection prognosis and showing increased morbidity and mortality from sepsis. The authors propose that T2D causes a functional immune deficiency that directly reduces immune cell function. T2D patients display diminished bactericidal clearance, increased infectious complications, and protracted sepsis mortality. This comprehensive review explores immune dysfunction including: metabolic regulation, inflammation, molecular pathways, cellular defects, cytokines, and immune modulatory therapies.

In this hypothesis and theory: “The Folate Cycle As a Cause of Natural Killer Cell Dysfunction and Viral Etiology in Type 1 Diabetes” Bayer and Fraker pose an interesting role of natural killer cells (NK) in T1D development. The authors describe a link between inefficient folate metabolism and poor antiviral responses from NK cells to the establishment of chronic viral infections. They hypothesize that defects in the folate cycle within genetically susceptible individuals could lead to immune dysfunction, create a permissive environment allowing for chronic or cyclical latent/lytic viral infections, a dampened NK response, and beta cell death.

The next hypothesis and theory article: “The Four-Way Stop Sign: Viruses, 12-Lipoxygenase, Islets, and Natural Killer Cells in Type 1 Diabetes Progression” by Semeraro et al. outlines a new idea that incorporates early antiviral immune effectors, NK cells, with proinflammatory processes involving 12-lipoxygenase occurring in the pancreatic beta cells. The authors hypothesize that the activation of NK cell lipoxygenase through viral infections could contribute to T1D initiation by affecting the normal balance of activating and inhibitory NK cell receptors, ultimately leading to autoimmunity and islet destruction.

Finally, the series concludes with a final hypothesis and theory: “Environmental Factors Contribute to β Cell Endoplasmic Reticulum Stress and Neo-Antigen Formation in Type 1 Diabetes” by Marre and Piganelli. This article summarizes the current knowledge regarding endoplasmic reticulum (ER) stress and protein post-translational modifications that can occur in islet beta cells, and it proposes a role for environmental factors in the breakdown of immunologic tolerance to beta cell antigens. The authors describe a number of factors including virus infection, dysglycemia, inflammation, chemical exposure, and ROS synthesis that can lead to ER stress in beta cells, neo-antigen formation, and priming autoreactive T cell responses.

## Author Contributions

JS and BF wrote the manuscript. All authors edited the final version.

### Conflict of Interest Statement

The authors declare that the research was conducted in the absence of any commercial or financial relationships that could be construed as a potential conflict of interest.

